# Expectations regarding the effectiveness of mask-wearing and pandemic fatigue: The experience in Japan

**DOI:** 10.1371/journal.pone.0321402

**Published:** 2025-05-14

**Authors:** Akihiro Kawase, Mototsugu Fukushige

**Affiliations:** 1 Faculty of Economics, Toyo University, Tokyo, Japan; 2 Graduate School of Economics, Osaka University, Osaka, Japan; University of Pretoria, SOUTH AFRICA

## Abstract

Even though mask-wearing was merely “recommended” in Japan during the COVID-19 pandemic, the mask usage rate was higher than that in other countries. This study conducted an econometric analysis to examine whether expectations regarding the effectiveness of mask-wearing was a motivating factor for Japanese people to wear masks during the prolonged pandemic. The results showed that, even when considering individual factors such as risk aversion, altruism, and social pressure, the motivation of wearing a mask for self-protection had a significant positive effect. This significance was maintained even in the second year of the survey, when pandemic fatigue was occurring. This was a distinctive feature, as the effects of other individual factors on pandemic fatigue had diminished. On the other hand, the motivation for wearing a mask to protect others did not have a significant effect in either year, which contrasted with the results for self-protection. These estimation results indicate that the route of wearing a mask for self-protection was continuously effective during the prolonged pandemic, and that promoting mask-wearing through this route is effective in inducing individual behavioral changes, even during a prolonged pandemic.

## Introduction

The spread of COVID-19 was a new infection not only in other countries in the world but also in Japan. Due to lack of knowledge and understanding of this disease, the Japanese government first proposed measures to avoid “the three Cs” (closed space, crowded place, close contact settings). Specifically, it was recommended to avoid ventilation, social distance, and face-to-face conversations. In connection with this, it was reported that wearing face masks was effective in preventing the spread of coronaviruses [1].

In response to such evidence, some countries, such as Australia, Brazil, China, France, and the United States, mandated mask-wearing [2]. Unlike other countries, in Japan from April 2020, wearing masks was just recommended and voluntary [3]. Nevertheless, the rate of mask use in Japan was higher than that in other countries [4]. Mask-wearing has been a long-standing practice in Japan since the 1918–1920 flu pandemic [5]. Of course, it is not clear whether this habit is that wearing a mask is an effective means to prevent infection, or that it is generally easy to see and is due to social pressure.

Two effects of mask-wearing have been identified: (1) the effect of not becoming infected oneself (filtration for wearer protection), and (2) the effect of not infecting others (source control) [6]. People might be motivated to wear masks to benefit themselves by not contracting the virus, or to benefit others by not transmitting the virus. Ueki et al. [7] reported that mask-wearing was more effective in reducing infection of the wearer, rather than reducing infection of others. We want to confirm the point out of Ueki et al. [7] and analyze how the effects of pandemic fatigue affect these motives.

Previous studies have found that the perceived efficacy of wearing a face mask for prevention is an essential predictor of mask-wearing behavior. For example, during the bird flu in Hong Kong [8], the 2009 swine flu outbreak in Hong Kong [9], and the early stages of COVID-19 in South Korea [10], the more people who perceived that mask-wearing was effective for preventing infection, the more likely they were to wear a mask as a self-protective behavior. As a behavior that could protect others, Lau et al. [11] showed that the efficacy of mask-wearing positively increased preventive behaviors in response to a hypothetical outbreak of human-to-human H5N1 transmission.

Regarding whether wearing a mask is for oneself or others, Rieger [12] conducted an online survey among the students and employees of a German university on perceptions of self-protection and protecting others when using face masks. His results showed that self-protection was an important factor for young people, while protecting others was an important factor for older people. By contrast, Asri et al. [13] surveyed the risk aversion and altruism of Swiss health-care workers and showed that older people were more motivated by selfish motives, whereas younger people were more motivated by altruistic motives. Thus, the results of existing studies conflict. It should be noted, however, that there is a difference in what “older” refers to: Rieger [12] refers to people aged 26 years and older, and Asri et al. [13] refer to people aged 45 years and older as “older”. These existing studies have yet to distinguish whether mask-wearing behavior is based on individual preferences or the prospects of preventing infection. Of course, other potential motivations for wearing masks during COVID-19 have shed some light on previous studies. People who wear masks tend to be more comfortable doing the same things that many people do, including being more conforming to social norms or pressures [14–17].

Building on previous research on the motivation behind mask-wearing, this study aims to clarify the impact of individual expectations regarding the effectiveness of wearing masks on mask-wearing behavior. The effectiveness of mask-wearing can be categorized into two effects: reducing one’s own risk of infection, and reducing the risk of infecting others. We analyzed whether expectations regarding each of these effects had an impact on mask-wearing behavior.

Our data were obtained from a nationally representative panel survey that tracked the same individuals’ mask-wearing behavior, perceived probability of infection, and preferences before and at 1 and 2 years after the pandemic. The data are microdata provided by the *Preference Parameters Study of Osaka University*. Based on these data, we can analyze how pandemic fatigue emerged in Japan. Pandemic fatigue in the second year of pandemic, which is characterized by a decline in willingness to adhere to recommended protective behaviors, has been observed in many countries as a result of prolonged compliance with COVID-19 restrictions [18]. In Japan, there has also been a decrease in preventive measures such as handwashing, mask-wearing, and social distancing [19]. The nature of pandemic fatigue is poorly understood, and the changes in motivations underlying such behavioral changes are unclear. Specifically, as will be described later, even in Japan, a small percentage of people stopped wearing masks all the time during the COVID-19 period, which can be considered pandemic fatigue.

Our analysis reveals that the motivation to wear masks for self -protection has a significant positive effect throughout the pandemic. The motivation of wearing a mask for self-protection has been maintained even in the second year of the survey, but the effects of other variables have disappeared in the second year of the survey. The latter change seems to be the effect of pandemic fatigue. On the other hand, the motivation to wear a mask to protect others had no significant effects in both years. This is the same as the result of Asri et al. [13]. Based on these estimates, we find the possibility that there is a route that continues to wear masks for self-protection, especially in the second year. Promoting masks through this route suggests that it is effective in inducing individual behavioral changes, even in long-term pandemics.

This paper is organized as follows. We first describe the background, data, and analytical methods as part of the materials and methods. Then, we present the results of the analysis. Finally, we discuss the results and consider remaining issues.

## Materials and methods

### Background

The first case of COVID-19 infection in Japan, involving a man who had traveled to Wuhan, China, was reported on January 16, 2020. On February 10, Japan recorded its first COVID-19-related death. In response to the escalating spread of infection, on February 27, the government requested nationwide temporary school closure for elementary, junior high, and high schools. On March 24, the government postponed the Tokyo Olympic Games scheduled for the summer of 2020. Additionally, on April 7, the government declared a state of emergency for seven prefectures, including Tokyo, and expanded this declaration to encompass all prefectures on April 16.

Japan’s mask policy was only a recommendation during the COVID-19 pandemic. On April 15, the Prime Minister’s Office recommended wearing masks in educational material titled “A Guide to Avoiding the Three Cs!”. Subsequently, the government implemented a program called “Abenomask,” named after Prime Minister Shinzo Abe, in which masks were provided free of charge to every family. Mask-wearing continued to be recommended until February 10, 2023, when the recommendation policy was replaced with a policy that mask-wearing should be left to the discretion of individuals.

### Data

The data utilized in this study were derived from microdata provided by the *Preference Parameters Study of Osaka University*, a survey that started in 2003 and has been conducted annually, with two interruptions. A two-stage stratified random sampling approach was employed. Initially, municipalities were categorized into 40 blocks based on regional location and population size, with targeted municipalities randomly selected from each block. Subsequently, residents’ names and addresses were randomly extracted from the *Basic Resident Book* (utilizing the voter registration list if the Register was inaccessible to the public). The survey employed visiting and placement (self-administered) methods, incentivizing respondents with gift vouchers. It specifically targeted individuals aged 20–69 years residing in Japan during the initial wave of data collection.

In this study, we used data from the 2021 and 2022 surveys because of the addition of questions on COVID-19 from the 2021 survey. The 2021 survey distributed questionnaires to 3,437 individuals, 2,733 of whom responded, representing a response rate of 79.5%. For the 2022 survey, the questionnaires were distributed to 5,841 individuals, 3,427 of whom responded, accounting for 58.7% of those surveyed. The 2021 survey was carried out from January to February 2021, and the 2022 survey from January to February 2022. For the analysis, we focused on the respondents who responded to the analysis for both years and analyzed the relationship between changes in mask wearing and variables such as individual attributes and the effect of pandemic fatigue.

The target variable for mask-wearing was constructed using the following question.

#### Mask-wearing.

The survey respondents were asked, “Please answer concerning your life in the first half of this January—I always wear a mask when I go out.” The respondents answered on a 5-point Likert scale from “(1) Doesn’t apply at all” to “(5) Applies exactly.” The 2021 survey also included a question regarding mask use as of 2020. The present study used mask-wearing as of 2020 as the baseline value.

The following two questions are the most important variables for predicting mask-wearing.

#### Expectation of self-protection.

The survey respondents were asked subjective probabilities of becoming infected using a figure from 0 to 100. First, the respondents were asked, “If you were leading the same way of life that you led before the COVID-19 pandemic, what would the percentage likelihood of you becoming infected in the next 12 months be?”

(0) Leading the same way of life that you led before the COVID-19 pandemic

Next, respondents were asked, “Assuming the case where you led the same way of life that you led before the COVID-19 pandemic while conducting only one out of the following countermeasures (1) to (5), what would the percentage likelihood of you becoming infected in the next 12 months in each case be?”

(1) I frequently wash and sanitize my hands.
(2) I always wear a mask when I go out.
(3) I keep ample distance when I talk to people.
(4) I do not take part in dining or drinking parties with people other than my family.
(5) I do not travel to other prefectures by public transport.

We constructed a variable for self-protection by subtracting (0) from (2). This variable can be interpreted as an additional increase in expectations regarding the effectiveness of mask-wearing.

#### Expectation of protecting others.

The survey respondents were asked subjective probabilities of transferring the virus to another person apart from their family using a value from 0 to 100. First, the respondents were asked, “Assuming that you had become infected with COVID-19 without showing any symptoms, what would the percentage likelihood of you transferring the virus to another person apart from your family be if you were leading the same way of life that you led before the pandemic?”

(0) Leading the same way of life that you led before the COVID-19 pandemic

Next, the respondents were asked, “Assuming the case where you led the same way of life that you led before the COVID-19 pandemic while conducting only one out of the following countermeasures (1) to (5), what would the percentage likelihood of you, as somebody infected without showing symptoms, transferring the virus to another person apart from your family in each case be?”

(1) I frequently wash and sanitize my hands.
(2) I always wear a mask when I go out.
(3) I keep ample distance when I talk to people.
(4) I do not take part in dining or drinking parties with people other than my family.
(5) I do not travel to other prefectures by public transport.

We constructed a variable for protecting others by subtracting (0) from (2). This variable can also be interpreted as an additional increase in expectations regarding the effectiveness of mask-wearing.

We also included the following variables in our analysis to explore additional potential motivations for mask-wearing.

#### Risk aversion.

The survey respondents were asked, “How high does the chance of rain have to be before you will bring an umbrella with you when you go out?” The respondents answered on a scale from 0 to 100.

#### Altruism.

The survey respondents were asked, “To what extent do you agree with each of the following statements? I feel happy when I do a good deed that I think benefits others (such as picking up trash in a park).” The respondents answered on a Likert scale from “(1) Completely agree” to “(5) Completely disagree.” For the analysis, we coded in reverse order so that higher values would indicate more altruism.

#### Social pressure.

The survey respondents were asked, “How true for you is the following statement? Behaving similarly to people around me makes me feel comfortable.” The respondents answered on a Likert scale from “(1) Particularly true” to “(5) Doesn’t hold true at all.” For the analysis, we coded in reverse order so that higher values would indicate more social pressure.

Additionally, we adopted variables for controlling the circumstances of the survey respondents.

#### Number of new cases and deaths.

Japanese prefectures announce the number of new infections and deaths daily, and the Ministry of Health, Labour and Welfare (MHLW) compiled and published these figures until May 2023. We use figures from the database compiled and published by the MHLW. If respondents made decisions regarding their behavior based on the previous week’s trend of newly infected cases, we used the average of the last week [20].

### Model

The estimation equation characterized by a latent variable used in this study was as follows:


$${Mask}_{i}^{*}=\beta_{0}+\beta_{1}Mas{k0}_{i}+({Expectation)}_{i}+(Preferenc{e)}_{i}+x_{i}^{\prime }\gamma +\varepsilon_{i}$$ (1)

where i represents an individual, the dependent variable ${Mask}_{i}^{*}$ is a latent variable of mask-wearing behavior, $Mas{k0}_{i}$ is a variable of mask-wearing as of 2020 to control for the habit of wearing masks to prevent hay fever and seasonal flu, $({Expectation)}_{i}$ denotes the expectation of the effectiveness of mask-wearing—this part is a linear combination of self-protection and protecting others, $(Preferenc{e)}_{i}$ represents individual preferences—this part is also a linear combination of the variables for risk aversion, altruism, and social pressure, and ${\text{x}}_{i}$ denotes the vector of covariates such as gender, age, household income, and presence or absence of a cohabitant. Following Haischer et al. [21], Looi [22], Yusof et al. [23] and other preceding research, we included these variables as covariates in a regression analysis. Unfortunately, we were unable to obtain data on the influence of authoritarian personality, political stance, or party affiliation, as pointed out by Chen et al. [24] and Magnus et al. [25], so we did not use these as explanatory variables. In addition, the numbers of new cases and deaths were included to represent the infection status of the region. $\varepsilon_{i}$ is an error term.

In this paper, we consider that we do not observe ${Mask}^{*}$ but rather an observed variable for Mask-wearing (${Mask}_{i}$) taking values 1–5 indicating increasing mask-wearing. Thus, we assume the relation between ${Mask}_{i}$ and ${Mask}_{i}^{*}$ as follows:


$${Mask}_{i}=j\text{ if }{/cut}_{j-1}\leq {Mask}_{i}^{*}<{/cut}_{j}\text{ for }j=1,\;\ldots ,\;5$$ (2)

where ${/cut}_{j}$ are unknown cut points to be estimated with ${/cut}_{0}=-\infty$ and ${/cut}_{5}=+\infty$.

We employed an ordered probit model for estimation of the model for ${Mask}_{i}$ by combining the equations (1) and (2) and introduce an assumption that the error term ($\varepsilon_{i}$) obeys the standard normal distribution. Method for estimation is the maximum likelihood estimation. As the scales of the independent variables of our interest differed, we standardized all variables to investigate their impact on mask-wearing [26].

## Results

### Descriptive statistics of the sample

Table 1 presents the descriptive statistics of the sample used in the analysis. Additionally, Tables 2 and 3 present the gender-specific descriptive statistics and the descriptive statistics for under the age of 60 group and aged 60 or older group, respectively. As we mentioned earlier, we focused on the respondents who responded to the analysis for both years and the sample was limited to respondents with no missing values for 2 years, resulting in 1,811 observations. Tables 2 and 3 reported their observations for each category. First, although not panel data, looking at the values of “Number of cases (prefecture level)” and “Number of deaths (prefecture level)” indicates a rapid decline in the pandemic in the second year compared with the first. Relevant to the purpose of this study, the manifestation of pandemic fatigue, the average value of “Mask-wearing” decreased slightly by just over 1%, from 4.809 to 4.751. As this is a change in the average value based on a Likert scale, it is difficult to grasp the specific meaning or consider what kind of change it represents based on this value alone. On the other hand, “Self-protection” increased slightly, by just over 1%, from 23.326 to 23.662, suggesting an increased understanding of mask-wearing. However, because this is also a change in the average value based on a Likert scale, it is difficult to grasp the specific meaning. In addition, age increased by 1 year and income increased slightly, which is likely because of age and years of service.

**Table 2:** Sample characteristics by gender.

Male					
Variables	Obs	Mean	SD	Min	Max
	2021				
Mask-wearing	869	4.701	0.786	1	5
Mask-wearing in 2020	869	2.983	1.710	1	5
Marginal benefit of self-protection	869	22.919	24.740	0	100
Marginal benefit of protecting others	869	29.237	27.585	0	100
Risk aversion	869	0.534	0.194	0	1
Altruism	869	3.811	0.690	1	5
Social pressure	869	2.895	0.864	1	5
Female	869	0	0	0	0
Age (years)	869	61.834	11.785	31	87
Household income (in million JPY)	869	6.201	4.027	0.5	20
Household income (ln)	869	1.609	0.702	-0.693	2.996
Cohabitant (yes = 1)	869	0.933	0.250	0	1
Number of cases (prefecture level)	869	25.261	16.045	0.777	63.557
Number of deaths (prefecture level)	869	0.383	0.397	0	1.629
	**2022**				
Mask-wearing	869	4.609	0.942	1	5
Mask-wearing in 2020	869	2.983	1.710	1	5
Marginal benefit of self-protection	869	23.955	25.795	0	100
Marginal benefit of protecting others	869	28.902	27.267	0	100
Risk aversion	869	0.541	0.201	0	1
Altruism	869	3.797	0.676	1	5
Social pressure	869	2.942	0.862	1	5
Female	869	0	0	0	0
Age (years)	869	62.834	11.785	32	88
Household income (in million JPY)	869	6.307	4.295	0.5	20
Household income (ln)	869	1.608	0.726	-0.693	2.996
Cohabitant (yes = 1)	869	0.926	0.261	0	1
Number of cases (prefecture level)	869	2.391	2.677	0	19.712
Number of deaths (prefecture level)	869	0.006	0.035	0	0.381
**Female**					
	**2021**				
Mask-wearing	942	4.909	0.520	1	5
Mask-wearing in 2020	942	3.241	1.702	1	5
Marginal benefit of self-protection	942	21.778	22.406	0	100
Marginal benefit of protecting others	942	25.557	25.213	0	100
Risk aversion	942	0.557	0.186	0	1
Altruism	942	3.910	0.650	1	5
Social pressure	942	2.956	0.882	1	5
Female	942	1	0	1	1
Age (years)	942	59.838	11.200	32	84
Household income (in million JPY)	942	5.799	3.759	0.5	20
Household income (ln)	942	1.533	0.726	-0.693	2.996
Cohabitant (yes = 1)	942	0.908	0.290	0	1
Number of cases (prefecture level)	942	24.891	16.265	0.777	63.557
Number of deaths (prefecture level)	942	0.377	0.386	0	1.629
	**2022**				
Mask-wearing	942	4.882	0.563	1	5
Mask-wearing in 2020	942	3.241	1.702	1	5
Marginal benefit of self-protection	942	23.392	24.167	0	100
Marginal benefit of protecting others	942	25.316	24.101	0	100
Risk aversion	942	0.543	0.189	0	1
Altruism	942	3.862	0.688	1	5
Social pressure	942	2.984	0.849	1	5
Female	942	1	0	1	1
Age (years)	942	60.838	11.200	33	85
Household income (in million JPY)	942	5.899	3.889	0.5	20
Household income (ln)	942	1.540	0.746	-0.693	2.996
Cohabitant (yes = 1)	942	0.904	0.294	0	1
Number of cases (prefecture level)	942	2.235	2.281	0	19.712
Number of deaths (prefecture level)	942	0.007	0.038	0	0.381

**Table 3:** Sample characteristics by age.

Young (Age is less than 60)					
Variable	Obs	Mean	SD	Min	Max
	2021				
Mask-wearing	817	4.874	0.536	1	5
Mask-wearing in 2020	817	3.072	1.715	1	5
Marginal benefit of self-protection	817	25.080	24.609	0	100
Marginal benefit of protecting others	817	27.913	26.487	0	100
Risk aversion	817	0.513	0.191	0	1
Altruism	817	3.807	0.703	1	5
Social pressure	817	3.010	0.899	1	5
Female	817	0.552	0.498	0	1
Age (years)	817	50.272	6.736	31	59
Household income (in million JPY)	817	7.445	4.092	0.5	20
Household income (ln)	817	1.832	0.655	-0.693	2.996
Cohabitant (yes = 1)	817	0.947	0.223	0	1
Number of cases (prefecture level)	817	25.983	16.534	0.777	63.557
Number of deaths (prefecture level)	817	0.384	0.375	0	1.629
	**2022**				
Mask-wearing	765	4.790	0.725	1	5
Mask-wearing in 2020	765	3.073	1.712	1	5
Marginal benefit of self-protection	765	26.527	25.448	0	100
Marginal benefit of protecting others	765	27.213	25.818	0	100
Risk aversion	765	0.515	0.193	0	1
Altruism	765	3.790	0.719	1	5
Social pressure	765	3.056	0.898	1	5
Female	765	0.556	0.497	0	1
Age (years)	765	50.678	6.552	32	59
Household income (in million JPY)	765	7.640	4.166	0.5	20
Household income (ln)	765	1.854	0.67	-0.693	2.996
Cohabitant (yes = 1)	765	0.949	0.220	0	1
Number of cases (prefecture level)	765	2.382	2.675	0	19.712
Number of deaths (prefecture level)	765	0.007	0.037	0	0.381
**Old (Age 60 year or older)**					
	**2021**				
Mask-wearing	994	4.756	0.757	1	5
Mask-wearing in 2020	994	3.154	1.705	1	5
Marginal benefit of self-protection	994	20.062	22.412	0	100
Marginal benefit of protecting others	994	26.838	26.394	0	100
Risk aversion	994	0.573	0.186	0	1
Altruism	994	3.908	0.640	2	5
Social pressure	994	2.859	0.846	1	5
Female	994	0.494	0.500	0	1
Age (years)	994	69.446	6.226	60	87
Household income (in million JPY)	994	4.798	3.274	0.5	20
Household income (ln)	994	1.354	0.690	-0.693	2.996
Cohabitant (yes = 1)	994	0.897	0.304	0	1
Number of cases (prefecture level)	994	24.316	15.808	0.777	63.557
Number of deaths (prefecture level)	994	0.377	0.404	0	1.629
	**2022**				
Mask-wearing	1,046	4.723	0.818	1	5
Mask-wearing in 2020	1,046	3.149	1.708	1	5
Marginal benefit of self-protection	1,046	21.567	24.390	0	100
Marginal benefit of protecting others	1,046	26.908	25.667	0	100
Risk aversion	1,046	0.562	0.194	0	1
Altruism	1,046	3.861	0.654	1	5
Social pressure	1,046	2.897	0.817	1	5
Female	1,046	0.494	0.500	0	1
Age (years)	1,046	69.926	6.481	60	88
Household income (in million JPY)	1,046	4.964	3.647	0.5	20
Household income (ln)	1,046	1.366	0.716	-0.693	2.996
Cohabitant (yes = 1)	1,046	0.890	0.313	0	1
Number of cases (prefecture level)	1,046	2.257	2.326	0	19.712
Number of deaths (prefecture level)	1,046	0.007	0.036	0	0.381

**Table 1:** Sample characteristics.

Variables	Mean	SD	Min	Max
	2021			
Mask-wearing	4.809	0.669	1	5
Mask-wearing in 2020	3.117	1.710	1	5
Marginal benefit of self-protection	22.326	23.555	0	100
Marginal benefit of protecting others	27.323	26.434	0	100
Risk aversion	0.546	0.190	0	1
Altruism	3.863	0.671	1	5
Social pressure	2.927	0.873	1	5
Female	0.520	0.500	0	1
Age (years)	60.796	11.524	31	87
Household income (in million JPY)	5.992	3.894	0.5	20
Household income (ln)	1.569	0.715	–0.693	2.996
Cohabitant (yes = 1)	0.920	0.271	0	1
Number of cases (prefecture level)	25.068	16.157	0.777	63.557
Number of deaths (prefecture level)	0.380	0.391	0	1.63
	**2022**			
Mask-wearing	4.751	0.781	1	5
Mask-wearing in 2020	3.117	1.710	1	5
Marginal benefit of self-protection	23.662	24.956	0	100
Marginal benefit of protecting others	27.037	25.724	0	100
Risk aversion	0.542	0.195	0	1
Altruism	3.831	0.683	1	5
Social pressure	2.964	0.855	1	5
Female	0.520	0.500	0	1
Age (years)	61.796	11.524	32	88
Household income (in million JPY)	6.094	4.093	0.5	20
Household income (ln)	1.573	0.737	–0.693	2.996
Cohabitant (yes = 1)	0.915	0.279	0	1
Number of cases (prefecture level)	2.310	2.479	0	19.712
Number of deaths (prefecture level)	0.007	0.036	0	0.381

The number of observations is 1,811 in each year. SD means standard deviation.

### Transition matrix of mask-wearing

Table 4 presents the transition matrices showing changes in mask-wearing. Panel A shows the transition matrix from 2020 to 2021, and Panel B shows that from 2021 to 2022. The data show the trend in Likert scale responses to the question “I always wear a mask when I go out.” Respondents answered this question on a 5-point Likert scale from “(1) Doesn’t apply at all” to “(5) Applies exactly,” with higher values indicating that the respondent wore a mask all the time.

As mentioned earlier, the 2021 survey included a question about mask usage in 2020. First, the results in Panel A of Table 4 show that 37% of the respondents always wore a mask when going out in 2020, and this value increased to 89% in 2021. This result indicates that while a certain number of people always wore masks when going out to prevent hay fever and seasonal influenza, the number of people who always wore masks increased significantly during the COVID-19 pandemic.

**Table 4:** Transition matrix of mask-wearing.

	Mask-wearing					
	1	2	3	4	5	Total
***Panel A. From 2020 to 2021***						
1	1.55	0.33	0.28	1.77	26.62	30.54
2	0.00	0.22	0.11	0.94	10.60	11.87
3	0.00	0.17	0.77	0.88	8.56	10.38
4	0.11	0.11	0.11	2.82	6.63	9.77
5	0.11	0.06	0.11	0.22	36.94	37.44
Total	1.77	0.88	1.38	6.63	89.34	100.00
***Panel B. From 2021 to 2022***						
1	0.44	0.00	0.00	0.22	1.10	1.77
2	0.11	0.22	0.11	0.28	0.17	0.88
3	0.00	0.17	0.33	0.39	0.50	1.38
4	0.06	0.06	0.88	2.43	3.20	6.63
5	2.15	0.44	0.55	4.14	82.05	89.34
Total	2.76	0.88	1.88	7.45	87.02	100.00

The number of observations is 1,811 each year. The values in each cell are in percent.

Next, Panel B of Table 4 shows the transition matrix for mask-wearing from 2021 to 2022. Overall, the proportion of people who always wore a mask when going out decreased slightly (by about 2.5%), from 89.34% in 2021 to 87.07% in 2022, suggesting that most people still wore masks all the time. However, focusing on those who answered “Applies exactly” (rated 5), there was a decrease of about 8.2%, from 89.34% to 82.05%, indicating that 8.2% of those who always wore masks the previous year stopped doing so, which is a non-negligible magnitude. Although the overall mask-wearing rate remained high, this change suggests that a certain proportion of people experienced pandemic fatigue.

### Estimation results

Before going into the analyses on the results of regression estimation, we should mention two things. One is the reason why we do not adopt some methods for panel data analysis. Another is the effects of the dropped respondents in the second year in comparing the estimation results for the first and second year to investigate pandemic fatigue.

The reason why we do not adopt the method for panel data estimation is as follows: If unobserved individual characteristics are taken into account using normal panel data analysis methods, it will also bring about serious problems in estimating the coefficients of variables representing expectations and preferences that are used as explanatory variables in regression analysis. For example, if we consider the data on (Marginal benefit of self-protection), even if the effect on mask-wearing in 2021 and 2022 was different, in other words, the coefficient in the regression analysis changed, if the value of this variable did not change, the effect of this variable on mask-wearing could not be identified when we also incorporate the considering unobserved individual characteristics, for example, introducing the difference between the two points in time or the coefficient of a dummy variable indicating an individual, which would have a significant impact on the estimation of the impact of pandemic fatigue. This problem introduces a crucial problem to the estimation of the effects of the coefficients about the expectations and preferences, which are the focus of this paper. Therefore, in this paper, we prioritize estimating the effect of variables that did not change at two points in time over estimating the effect from unobserved individual attributes, and adopt a cross-sectional analysis.

As for the effects of the dropped respondents in the second year in comparing the estimation results for the first and second year to investigate pandemic fatigue, looking ahead to the results of the likelihood ratio test regarding the significance of the changes in the estimated results when the deleted observations are added, the result of the likelihood ratio test comparing the estimated results when the dropped observations are added to the selected model in 2021 in Table 5 with the estimated results when the dummy variable and the cross terms of the dummy variables and selected variables to allow the coefficients of the deleted observations to differ from those of the observations that were not dropped are added is 8.43. Since the statistic of this test asymptotically follows a chi-square distribution with 9 degrees of freedom, the p-value is calculated to be 0.4917, which is not statistically significant. Therefore, it is suggested that the deleted observations do not provide statistically significant results in the comparison of the results for 2021 and 2022.

**Table 5:** Regression results of ordered probit with standardized variables.

	2021		2022	
	Full	Selected	Full	Selected
Mask-wearing in 2020	0.284^**^	0.282^**^	0.145^**^	0.144^**^
	(0.041)	(0.041)	(0.039)	(0.038)
Marginal benefit of self-protection	0.176^**^	0.201^**^	0.211^**^	0.196^**^
	(0.051)	(0.049)	(0.051)	(0.047)
Marginal benefit of protecting others	0.062		–0.040	
	(0.045)		(0.043)	
Risk aversion	–0.003		0.036	
	(0.041)		(0.038)	
Altruism	0.114^**^	0.117^**^	0.032	
	(0.039)	(0.039)	(0.038)	
Social pressure	0.117^**^	0.113^**^	0.057	
	(0.042)	(0.042)	(0.040)	
Female	0.354^**^	0.348^**^	0.326^**^	0.336^**^
	(0.049)	(0.048)	(0.041)	(0.041)
Age	1.066^**^	1.048^**^	–0.053	
	(0.369)	(0.367)	(0.348)	
Age squared	–1.156^**^	–1.135^**^	–0.005	
	(0.367)	(0.365)	(0.347)	
Household income (ln)	0.065	0.091^*^	0.004	
	(0.046)	(0.043)	(0.044)	
Cohabitant	0.059		0.021	
	(0.038)		(0.039)	
Number of cases	0.004		–0.051	
	(0.047)		(0.036)	
Number of deaths	0.017		–0.050	
	(0.046)		(0.036)	
/cut1	2.508^*^	2.280^*^	–1.134	–1.278^**^
	(1.063)	(1.029)	(0.947)	(0.090)
/cut2	2.700^*^	2.471^*^	–1.008	–1.152^**^
	(1.056)	(1.022)	(0.946)	(0.089)
/cut3	2.913^**^	2.682^**^	–0.797	–0.943^**^
	(1.052)	(1.018)	(0.948)	(0.089)
/cut4	3.492^**^	3.259^**^	–0.282	–0.433^**^
	(1.054)	(1.018)	(0.950)	(0.091)
Observations	1,811	1,811	1,811	1,811
Pseudo R-squared	0.112	0.109	0.0680	0.0617
AIC	1,491	1,485	1,823	1,815

The dependent variable is mask-wearing. Standardized coefficient and robust standard errors are reported. Significance levels:

* p < 0.05,

** p < 0.01.

Table 5 shows the estimation results of the model: combination of the equations (1) and (2), in which we reported the estimated standardized coefficients with robust standard errors. We started estimation with the complete sets of candidate independent variables. Then, we selected the combination of independent variables that minimized the Akaike information criterion (AIC) for optimal model selection. Hence, in this analysis, we applied the AIC-minimizing method for model selection as a linear model by dropping explanatory variables in order of t-values. The columns for “Full” mean the estimated results with the complete set of independent variables for 2021 and 2022, and columns for “Selected” mean the results using AIC-minimizing for 2021 and 2022.

Next, we focus on the variables of self-protection and protecting others, which are the primary focus of this paper. Regarding self-protection, individuals who believed that mask-wearing was highly effective in protecting themselves from infection exhibited higher mask-wearing rates during the pandemic, significantly contributing to mask-wearing rates in both years. The standardized coefficients were 0.201 in 2021 and 0.196 in 2022, showing a slight downward trend, but remaining significantly positive throughout the period. On the other hand, protecting others did not significantly impact mask usage in either year. This suggests that even if individuals believe that mask-wearing is highly effective in preventing the infection of others, it does not necessarily translate into higher personal mask-wearing behavior.

The estimation results for the other variables are as follows. Risk aversion was not statistically significant in any year. Altruism was shown to have a positive impact only in 2021, with more altruistic people tending to wear masks, but that impact was no longer observed in 2022. Social pressure, i.e., those who feel comfortable behaving similarly to others, positively affected mask use in 2021, but this effect was not observed in 2022. Additionally, the estimation results indicate that mask-wearing as of 2020 was positively associated with mask-wearing during the COVID-19 pandemic. However, the effect was smaller in 2022 than in 2021. Females were more likely than males to wear masks in both years. An age effect was observed only in 2021, when the estimate of the single term was significantly positive, and its squared term was negative; however, this effect was not observed in 2022. Household income was statistically significant in 2021, but this effect was not observed in 2022. Those living with a cohabitant were not significant in either year. The numbers of new cases and deaths had no statistically significant effect. The estimation results indicate that the decision to wear a mask was not based on regional infection status.

### Results by age group

Checking the robustness of the result above, we conducted an analysis by splitting the sample at age 60 years. The reason we separated the sample at age 60 years is that COVID-19 infection is known to have higher severe disease and fatality rates in older adults, with dramatic increases seen after the age of 60 years [27]. A rapid increase in mortality rates among those aged 60 years and over has also been observed in China [28]. The results are reported in Table 6 and the selected variables are similar to those in Table 5.

**Table 6:** Regression results of ordered probit with standardized variables by age groups.

	Young		Old	
	Full	Selected	Full	Selected
	2021			
Mask-wearing in 2020	0.234^**^	0.229^**^	0.323^**^	0.318^**^
	(0.065)	(0.066)	(0.052)	(0.051)
Marginal benefit of self-protection	0.224^**^	0.193^*^	0.172^**^	0.180^**^
	(0.082)	(0.082)	(0.061)	(0.061)
Marginal benefit of protecting others	–0.050		0.124^*^	0.121^*^
	(0.065)		(0.061)	(0.061)
Risk aversion	–0.025		0.008	
	(0.068)		(0.050)	
Altruism	0.131^*^	0.133^*^	0.102^*^	0.105^*^
	(0.064)	(0.063)	(0.050)	(0.050)
Social pressure	0.205^**^	0.209^**^	0.058	
	(0.066)	(0.066)	(0.053)	
Female	0.397^**^	0.403^**^	0.344^**^	0.347^**^
	(0.080)	(0.080)	(0.063)	(0.063)
Age	0.162^*^	0.161^*^	–0.098	–0.095
	(0.069)	(0.070)	(0.059)	(0.056)
Household income (ln)	0.118	0.128^*^	0.014	
	(0.067)	(0.064)	(0.058)	
Cohabitant	0.031		0.079	0.086
	(0.059)		(0.052)	(0.048)
Number of cases	–0.050		0.033	
	(0.079)		(0.057)	
Number of deaths	0.044		–0.003	
	(0.077)		(0.058)	
Observations	817	817	994	994
Pseudo R-squared	0.136	0.134	0.0962	0.0947
AIC	511.4	502.8	996.8	988.4
	**2022**			
Mask-wearing in 2020	0.137^*^	0.137^*^	0.146^**^	0.145^**^
	(0.064)	(0.063)	(0.049)	(0.049)
Marginal benefit of self-protection	0.249^**^	0.268^**^	0.182^**^	0.148^*^
	(0.080)	(0.069)	(0.065)	(0.062)
Marginal benefit of protecting others	–0.001		–0.067	
	(0.075)		(0.053)	
Risk aversion	0.043		0.037	
	(0.061)		(0.047)	
Altruism	0.078		–0.006	
	(0.063)		(0.047)	
Social pressure	0.104		0.020	
	(0.066)		(0.047)	
Female	0.307^**^	0.308^**^	0.354^**^	0.355^**^
	(0.065)	(0.065)	(0.055)	(0.054)
Age	–0.076		–0.023	
	(0.063)		(0.051)	
Household income (ln)	0.047		–0.020	
	(0.062)		(0.054)	
Cohabitant	–0.037		0.060	
	(0.054)		(0.053)	
Number of cases	–0.075		–0.024	
	(0.058)		(0.045)	
Number of deaths	–0.026		–0.067	–0.070
	(0.078)		(0.037)	(0.038)
Observations	765	765	1,046	1,046
Pseudo R-squared	0.0801	0.0673	0.0636	0.0605
AIC	687.1	678.2	1158	1145

The dependent variable is mask-wearing. Standardized coefficient and robust standard errors are reported. Significance levels:

* p < 0.05,

** p < 0.01.

As for the variables of self-protection and protecting others, which are the primary focus of this paper, self-protection was positive and significant for both young and old samples in 2021 and 2022. In 2021, the standardized coefficients for the young and old samples were similar, at 0.193 and 0.180, respectively. In 2022, the standardized coefficient for the old sample became smaller than that in 2021, at 0.148, but the standardized coefficient for the young sample was larger than that in 2021, at 0.268. However, all the estimated coefficients were positive and significant. As for protecting others, in Table 6, the coefficient of protecting others in the old sample in 2021 was positive and significant. The standardized coefficient was 0.121, smaller than the marginal benefit of self-protection, which was 0.180. This result is similar to Asri et al. [13], but the variable was not selected in 2022. Although no such analysis has been conducted in Japan, as Adaryukov et al. [29] points out, it may be related to differences in perceptions of oneself and others regarding mask wearing, or differences in perceptions that have emerged over time. However, as can be seen in Table 4, in Japan, where nearly 90% of people answered that they always wear a mask, it is expected that the impact of these differences in perception on mask wearing rates is small.

Altruism was positive and significant only in 2021, with standardized coefficients of 0.133 for the young and 0.105 for the old sample, which were larger for the young. On the other hand, social pressure in 2021, which was positive and significant in Table 5, was positive and significant only for the young sample in Table 6. The standardized coefficient was 0.209, the highest value for young adults’ motivation to wear a mask in 2021. All the selected variables above in the result for 2021 are not significant in the result for 2022.

## Discussion

This study conducted an ordered probit analysis to examine whether expectations regarding the effectiveness of mask-wearing and other factors were motivating factor for Japanese people to wear masks during the prolonged pandemic. From the viewpoint of the selected variables in regression analysis, only the variables of self-protection and the dummy variable for females were selected both in 2021 and 2022 results, the variables of protecting others, altruism and the variable for social pressure are selected only in the 2021 result and other variables, i.e., the variables for risk aversion, age, dummy variable for those living with a cohabitant, the numbers of new cases and deaths were not selected in any years’ results. Focusing on the perspective of expectations regarding the effectiveness of mask-wearing and the emergence of pandemic fatigue through a comparison of the results for 2021 and 2022, we can draw the following conclusions. While the coefficient for the marginal benefit of self-protection, which was estimated to be statistically significant and positive in both years, was slightly smaller in 2022 than in 2021, but the significant positive effect was maintained. This contrasts with the fact that other variables were not selected as explanatory variables in the results for 2022, when pandemic fatigue seemed to have emerged. In other words, these variables had a positive effect on mask-wearing in the short term but lost their effectiveness as a result of pandemic fatigue. This result is robust when we separate the sample by age groups.

Such manifestations of pandemic fatigue suggest that, to maintain the effectiveness of infection prevention measures such as mask-wearing, it is increasingly important to promote expectations of their effectiveness as a pandemic prolongs. Moreover, if pandemic fatigue occurs in infection prevention behaviors besides mask-wearing, individual differences in infection prevention behaviors may be only short term, and the effects of individual attributes may diminish over the long term because of pandemic fatigue. This suggests that it is desirable to strengthen public awareness campaigns to maintain and enhance infection prevention activities. Of course, even if the mask-wearing rate reaches 100% as a result of individuals overestimating the effectiveness of masks, it is important to note that the probability of becoming infected or infecting others still exists, even while wearing a mask. Of course, as Sharma et al. [30] point out, the effectiveness of different types of masks in preventing disease is also not within the scope of this paper’s analysis. Therefore, we cannot expect more than the actual effect of mask-wearing on infection prevention, which is the ultimate goal of the policy.

Several limitations of this study should be acknowledged. First, our mask-wearing measures were subjective and the responses were self-reported. In addition, in the 2021 survey, the respondents were asked to recall their mask-wearing from 2020. This is a standard limitation in such studies. Unfortunately, we do not have available observational data on actual mask wearing rates, as used by Seresirikachorn et al. [31] for Thailand. Second, since April 2021, the Japanese government has preferentially distributed COVID-19 vaccines to older adults [32]. Our results showed that the tendency for mask-wearing rates to decline after a certain age may indicate substitution between vaccination and mask-wearing. Unfortunately, the data set does not contain vaccination information, which we cannot reveal from the analysis. Third, the data used in this study were based on a survey conducted in only one country, Japan. Further research is needed to determine whether the results can be generalized to other countries and regions. Fourth, it would be interesting to know how social networks such as Twitter, as analyzed by Hopfer et al. [33] and Lang et al. [34], have influenced mask wearing, including the impact on pandemic fatigue, but detailed data are not currently available.
